# Enhancing cancer immunotherapy with photodynamic therapy and nanoparticle: making tumor microenvironment hotter to make immunotherapeutic work better

**DOI:** 10.3389/fimmu.2024.1375767

**Published:** 2024-04-05

**Authors:** Jayalakshmi Thiruppathi, Veena Vijayan, In-Kyu Park, Shee Eun Lee, Joon Haeng Rhee

**Affiliations:** ^1^ Department of Microbiology, Chonnam National University Medical School, Hwasun-gun, Jeonnam, Republic of Korea; ^2^ Clinical Vaccine R&D Center, Chonnam National University, Hwasun-gun, Jeonnam, Republic of Korea; ^3^ Combinatorial Tumor Immunotherapy Medical Research Center (MRC), Chonnam National University Medical School, Hwasun-gun, Jeonnam, Republic of Korea; ^4^ National Immunotherapy Innovation Center, Hwasun-gun, Jeonnam, Republic of Korea; ^5^ Department of Biomedical Sciences, Chonnam National University Medical School, Hwasun-gun, Jeonnam, Republic of Korea; ^6^ Department of Radiology, Biomolecular Theranostics (BiT) Laboratory, Chonnam National University Medical School, Hwasun-gun, Jeonnam, Republic of Korea; ^7^ Department of Pharmacology and Dental Therapeutics, School of Dentistry, Chonnam National University, Gwangju, Republic of Korea

**Keywords:** photodynamic therapy, cancer immunotherapy, immune checkpoint inhibitors, immunogenic cell death, tumor microenvironment

## Abstract

Cancer immunotherapy has made tremendous advancements in treating various malignancies. The biggest hurdle to successful immunotherapy would be the immunosuppressive tumor microenvironment (TME) and low immunogenicity of cancer cells. To make immunotherapy successful, the ‘cold’ TME must be converted to ‘hot’ immunostimulatory status to activate residual host immune responses. To this end, the immunosuppressive equilibrium in TME should be broken, and immunogenic cancer cell death ought to be induced to stimulate tumor-killing immune cells appropriately. Photodynamic therapy (PDT) is an efficient way of inducing immunogenic cell death (ICD) of cancer cells and disrupting immune-restrictive tumor tissues. PDT would trigger a chain reaction that would make the TME ‘hot’ and have ICD-induced tumor antigens presented to immune cells. In principle, the strategic combination of PDT and immunotherapy would synergize to enhance therapeutic outcomes in many intractable tumors. Novel technologies employing nanocarriers were developed to deliver photosensitizers and immunotherapeutic to TME efficiently. New-generation nanomedicines have been developed for PDT immunotherapy in recent years, which will accelerate clinical applications.

## 1 Introduction

### 1.1 The cancer immunotherapy era arrived

Immunotherapy has changed the cancer treatment landscape, where long-term survival and durable cures are reported even for heterologous metastatic solid tumors (1). During the past decade, immunotherapy reshaped the cancer therapy paradigm with the successes of checkpoint blockade, cell therapeutics, and anti-tumor monoclonal antibody drugs (2, 3). Especially, checkpoint inhibitors including anti-PD-1, anti-PD-L1, and anti-CTLA4 blocking inhibitory signaling in effector anti-tumor T cells are significantly effective in treating renal cell carcinoma, melanoma, non-small cell lung carcinoma, head and neck cancers, hepatocellular carcinoma, triple-negative breast cancer, and others (2, 4, 5).

### 1.2 But cancer immunotherapy is not for all

**Figure 1:**
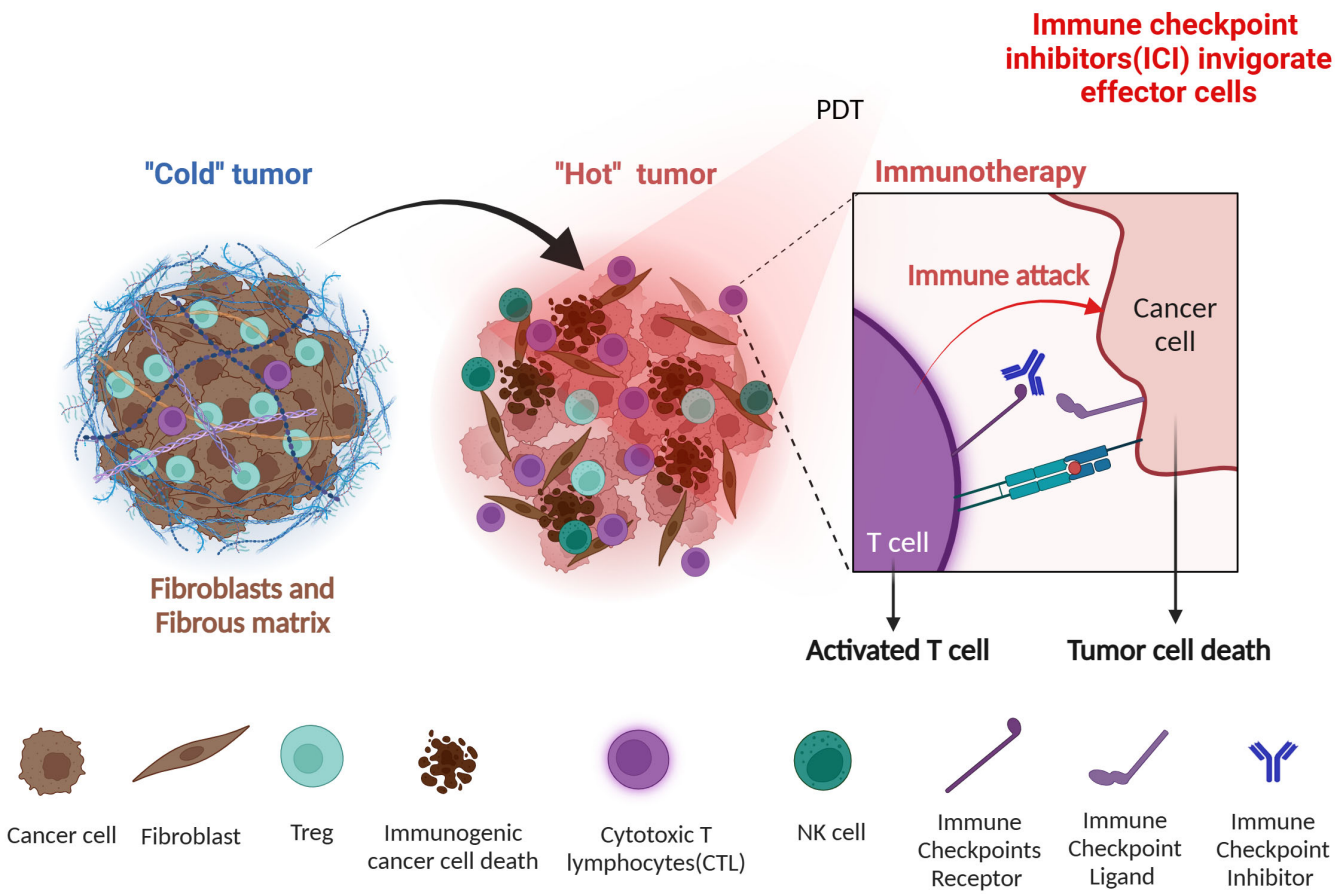
PDT contributes to turning cold tumors into hot. Cancer cells modulate their microenvironment to promote immune escape and increase immunosuppressive cell infiltration to make “cold” TME, compromising the efficacy of immunotherapy. By inducing immunogenic cell death, PDT switches the immunosuppressive TME toward “hot,” facilitating the recruitment of immune cells and subsequent tumor-specific immune responses that could be boosted by immunotherapeutic such as ICIs. Figure generated with Bio Render.

To our disappointment, most patients harbor immune “cold” tumors poorly responding to the above-mentioned checkpoint therapies. The immunosuppressive tumor microenvironment (TME) resists immune reversal by the checkpoint blockade due to its multimodal nature encompassing suppressive cytokines, lack of antigen presentation, T cell exhaustion, hostile metabolic states, and nutrient deprivation (5). Based upon the insights obtained through studying mechanisms of resistance to cancer immunotherapy, efforts are underway to overcome the current limitations. To improve the efficacy of cancer immunotherapies, ICIs (anti-PD-1/PD-L1 and anti-CTLA-4) and newer monoclonal antibody drugs(inhibiting TIGIT, TIM-3, LAG-3, VISTA, NKG2A and others) are tried in combination (6, 7). Additionally, ICIs are also used to enforce the effects of cell therapies (adoptive cell therapy, CAR T/NK therapy, TCR T therapy) and therapeutic cancer vaccines (8). As **Figure 1** summarizes, a key to success is that the combination strategies should be generally designed with the consideration of converting immunologically “cold” TME into “hot” status thereby enhancing the efficacies of immunotherapeutic treatments (9, 10). Such combination approaches, especially those inducing immunogenic cell death (ICD), are crucial for breaking the immunosuppressive barriers established by tumors, which continually alter to evade immune surveillance (11).

### 1.3 TME matters!

TME is complex and continuously evolving. In addition to stromal cells, fibroblasts, and endothelial cells, the TME contains a diverse array of innate and adaptive immune cells. Cancer cells secrete various cytokines, chemokines, and growth factors to fabricate immune evading TME. Growing evidence suggests that the innate immune cells (macrophages, neutrophils, dendritic cells, innate lymphoid cells, myeloid-derived suppressor cells, and natural killer cells) as well as adaptive immune cells (T cells and B cells) infiltrating the TME paradoxically contribute to tumor progression and metastasis (12). In the case of the established large melanoma model, where immunosuppressive TME had been heavily established, making the TME responsive to immunotherapy necessitated a complex combination of immunotherapeutic strategies. The research highlights the necessity of a multifaceted approach, incorporating a tumor-antigen-targeting antibody, a modified interleukin-2 with extended half-life, anti-PD-1, and a potent T cell vaccine, to effectively mobilize both innate and adaptive immune mechanisms against the tumor (13). The treatment not only facilitated the infiltration of these immune cells into the tumor but also enhanced the inflammatory cytokine milieu, which led to antigen spreading. This orchestrated response resulted in the effective eradication of established large tumors by leveraging the body’s endogenous immune capabilities to break the formidable immunosuppressive TME. If any of the four immunotherapeutic components were absent, the anti-tumor effect was seriously compromised, suggesting both innate and adaptive immune cells in the TME should have been activated (13). Modulating diverse cell populations contributing to immunosuppression by biological tools should require a multimodal approach that may claim high cost and risk of side effects caused by combined therapeutics.

### 1.4 Break the immunosuppressive TME abruptly

Radiotherapy (RT) is a local ablative physiotherapy that kills cells and modifies stromal connective tissue (14). In addition to killing tumor cells by ionizing radiation, RT also affects other components in TME, such as immune cells, cancer-associated fibroblasts, endothelial cells, etc. The bystander effects of RT employ molecular signals from irradiated cells affecting adjacent non-irradiated cells and tissues (15). The RT bystander effect brings abscopal tumor suppression through the induction of tumor-specific systemic immune response (16). By RT, TME converts from immunosuppressive to immunostimulatory status by (*1*) increased immune cell infiltration, (*2*) innate and adaptive immune response activation, (*3*) enhanced existing T cell responses, (*4*) increased immunogenicity of tumor cells, (*5*) induction of neoantigen-induced immune responses resulting in epitope expansion, and (*6*) changed cytokine/chemokine context (14). In this regard, RT-mediated tumor suppression and improved prognosis were more dominantly attributed to the RT-induced immune responses rather than the intrinsic radio-sensitivity of the tumor cells (17). In addition to the suppression of tumor growth at the irradiated site, RT-induced anti-tumor immune response effectively suppresses metastasis and tumors in remote sites, which is called “abscopal effect.” The abscopal effect is tightly related to RT-induced ICD of cancer cells (18). In addition, ICD is often referred to as immunogenic apoptosis since it is usually induced by apoptosis. There is also evidence that antitumor immunity may be mediated by several non-apoptotic cell death mechanisms (19). In necroptosis, for example, programmed cell death is combined with the release of intracellular contents or with priming and activating effector T cells to enhance antitumor immunity (20). In addition, ferroptosis has been recognized as a novel mechanism of cell death resulting from the accumulation of lipid peroxides by iron ions (21). Tumor cell-intrinsic events driven by DNA damage play a central role for the immunomodulatory actions of RT (22, 23). DNA damage and altered gene transcription due to radiation would result in cancer neoantigen expression (24).

### 1.5 Drawbacks and limitations of RT

RT is an efficient way of modulating TME to be more responsive to immunotherapy. However, RT has drawbacks and limitations. RT requires a well-equipped facility with costly irradiators and skillfully trained personnel, at high costs. Irradiation would cause collateral destruction of normal cells and tissues in the beam path. Though new technologies were devised to be less traumatic, side effects are still observed, including late psychoneurological dysfunctions (25, 26). Radiation activates a damage repair cascade in normal tissues. This cascade initiates with the DNA damage response accompanying apoptosis and cellular senescence (27) and is followed by proinflammatory and fibrogenic cytokine cascade inducing inflammation and excessive collagen deposition (28). To worsen the tissue traumatic situation, RT induces so-called ‘bystander’ injury to non-irradiated cells (29). Immunotherapy also accompanies a vast spectrum of side effects ranging from mild inflammation to severe life-threatening side effects, which may aggravate RT-mediated tissue injury.

### 1.6 Photodynamic therapy is a more amenable alternative to RT

Among local ablative physiotherapies, photodynamic therapy (PDT) kills with a mechanism similar to RT. It is based on the local or systemic application of a photosensitive compound - the photosensitizer (PS), specifically accumulated in target tissues. The PS has an absorption wavelength between 650 and 850 nm, which has the good tissue penetration and provides sufficient energy for generating excited states capable of reacting with molecular oxygens (30). The photoactivated PS leads to the generation of reactive oxygen species (ROS) from molecular oxygens. Generated ROS exerts cytotoxicity to the cells where the photosensitizer accumulates. The main ROS generated by PDT is singlet oxygens (^1^O_2_), formed by energy transfer from the activated photosensitizer. Radical species, such as the superoxide ion (O_2_^•–^) and hydroxyl radical (OH^•^), developed by electron transfer reactions, are also generated like the ionizing radiation. These ROS are very short-lived, reacting with biomolecules within a micron of generation focus, which should minimize collateral damage to bystander cells. Building upon the action of PDT, current research is extending into the realm of nanotechnology to further enhance therapeutic efficacy, especially in the field of combination therapy. By employing nanoparticles (NPs) for the delivery of immunomodulatory molecules, it became possible to bolster the effectiveness and diminish toxic side effects. Similarly, NPs could be engineered to capture tumor-derived antigens released after PDT, enabling the development of advanced NP-based *in situ* vaccines (31). These vaccines aim to amplify T cell responses through the antigen-adjuvant co-delivery, facilitating the activation of DCs and multiple antigen presentation. Moreover, nanotechnology introduces novel approaches such as *in situ* vaccination with artificial antigen-presenting cells or the placement of immune depots near tumor sites to enhance immunotherapeutic efficacy (32). For the clinical application, selective delivery of PSs to TME is the key to success. During the last decade, quantum progress was achieved in the nanotechnology field. The delivery of PS nanocarriers has significantly improved the efficacy and safety of PDT. The use of nanocarriers enables targeted delivery to focused lesions with high selectivity, which will contribute to dose reduction and improved safety. The anti-tumor effect of PDT is mediated by a combination of three mechanisms: (*1*) the direct cytotoxic effect on the cancer cells, (*2*) the destruction of the tumor blood vessels, and (*3*) the induction of anti-tumor immunity (30). In this review, we will examine the integration of immunotherapy, photodynamic therapy (PDT), and nanotechnology in cancer treatment. We aim to highlight how these approaches collectively enhance therapeutic efficacy, reduce side effects, and personalize care. By focusing on the advancements in targeted delivery and the modulation of the tumor microenvironment, we will underscore the potential of these innovative strategies to transform the landscape of cancer therapy, setting a new standard for patient care.

## 2 PDT and anti-cancer immunity

The history of PDT application to treat oncologic diseases goes back many years (33). The anti-tumor effects of PDT occur in a sequence of three mechanistically linked events. The first event happening to cancer cells is cell death by apoptosis, necroptosis, autophagy, and/or proptosis. A blood supply block and resulting hypoxia accompanying tumor vasculature destruction follow the acute cancer cell death, which further eliminates viable cancer cells (34, 35). These two events activate/release DAMPs in TME, which prime the innate and adaptive immune system against primary and abscopal tumors (36, 37).

### 2.1 PDT and requirements for ICD to induce better tumor suppression

Not all cancer cell-killing therapies induce ICD. ICD is reported to be induced by diverse physicochemical stresses encompassing from chemotherapeutics to PDT (38). Not surprisingly, only a few chemotherapeutic agents can cause the ICD of cancer cells. Moreover, ICD-inducing activities of any given chemotherapeutic cannot be predicted by the structure-function relationship. The gold standard approach for determining whether a cytotoxic intervention provokes *bona fide* ICD relies on vaccination experiments involving murine cancer cells and syngeneic, immunocompetent mice. Additionally, the ability of a specific therapeutic modality to induce ICD can be inferred by testing its antineoplastic effects on tumors established in immunocompetent *versus* immunodeficient hosts (39). This same principle should also be applied to PDT. Different PSs and activating light sources would divergently act in inducing ICD. ICD is defined with a unique response pattern of inducing organellar and cellular stresses, which eventually results in cell death accompanied by the exposure, active secretion, or passive release of numerous DAMPs (40). The spatiotemporally defined presentation of DAMPs during ICD and their recognition by specific PRRs expressed on antigen-presenting cells initiates a cascade of reactions that activate both innate and adaptive immune responses (40, 41). DAMPs liberated from cells undergoing ICD include endoplasmic reticulum (ER) chaperones such as calreticulin (CRT) and heat-shock proteins (HSPs), which are exposed on the cell surface, the non-histone chromatin-binding protein high-mobility group box 1 (HMGB1), the cytoplasmic protein annexin A1 (ANX1), and ATP liberated from dying cells, as well as *de novo* synthesized type I interferons (IFNs) (42–44). The DAMP recognition by PRRs expressed in innate and adaptive immune cells results in chemoattraction, homing, activation, and/or maturation of effector cells, which collectively contribute to tumor suppression (41). Any ICD condition that induces more robust cross-presentation of tumor antigens to CD8^+^ CTLs will improve therapeutic outcomes (45). Though similarly enhance anti-tumor immune responses, not all ICD inducers activate the same stress responses and elicit the same downstream signaling pathways (46). Different ICD inducers elicit overlapping biomarker signatures. Chemotherapeutics elicits the most diverse ICD biomarkers: ATP, ANX1, CRT, HMGB1, type I interferon, IL-1β, IL-17, and CXCL10. RT and PDT elicit overlapping biomarkers: ATP, HSP, CRT, HMGB1, and type I interferon (38). CRT exposed on the plasma membrane of cancer cells undergoing ICD serves as an “eat-me” signal that facilitates the engulfment of dying cells by antigen-presenting cells (30, 47). HSPs play important roles in the cellular stress response by detecting and re-folding misfolded proteins. During the ICD process, HSP70 and HSP90 are translocated into the plasma membrane and act as an “eat me” signal. Cell surface HSPs are recognized by receptors (such as CD91, LOX1, and CD40) on antigen-presenting cells, which, in turn, mediates the uptake of dying cells and the cross-presentation of tumor antigens to CD8^+^ T cells. Alternatively, HSP can also be recognized by the CD94 receptor of NK cells, which can directly kill cancer cells (30). PDT-mediated cell killing efficiency and ICD induction appear to be dependent upon PS chemistry, the light dosage, and the oxygenation status of the target tissue. The intracellular localization of the PS would be a determining factor. Typically, hydrophobic PSs accumulate in the membranes of endoplasmic reticulum, Golgi apparatus, and mitochondria, whereas hydrophilic PSs are more readily observed in the endocytic pathway. The pattern of accumulation of PS strongly impacts the stress adaptation and ICD process. For instance, the PS hypericin, when used under specific light and oxygenation conditions, has been observed to induce a pronounced ICD response by effectively triggering DAMP release (48). Conversely, when the light dosage is suboptimal or the tissue is hypoxic, the ICD effect may be significantly attenuated (49). The oxidative damage to the endoplasmic reticulum/Golgi apparatus and mitochondria is generally more lethal (50). In addition to ICD-associated DAMP release and subsequent production of proinflammatory cytokines in TME, PDT-generated ROS was reported to directly modulate tumor-associated macrophages (TAMs) to make the TME more responsive to immunotherapy. The ICD-inducing potential of PDT is not uniform across all PSs and treatment conditions. This variability necessitates a comparative analysis of different PDT protocols to determine the most efficacious combination for ICD induction. A protocol employing hypericin might excel in generating a strong antigenic response through ICD, whereas others may require adjunctive strategies to achieve comparable immunogenicity (51). The combination of PDT with other therapeutic modalities, such as chemotherapy or immune checkpoint inhibitors (ICI), can further augment the antigenic landscape. Chemotherapeutic agents such as doxorubicin have been reported to synergize with PDT to enhance the ICD effect, potentially due to the increased oxidative stress and subsequent DAMP release (52). Similarly, the blockade of immune checkpoints in conjunction with PDT can amplify the immune response to the antigens generated through ICD (52). The immunogenic potential of antigens generated through PDT-induced ICD is not merely a consequence of DAMP release but also the qualitative changes in the antigenic peptides presented by major histocompatibility complex (MHC) molecules on the surface of dying tumor cells. The modification of tumor antigens and the upregulation of MHC molecules are influenced by the intracellular oxidative milieu post-PDT, which can be optimized through protocol manipulation (53). The comparison of PDT protocols in the context of ICD and antigen generation reveals that a tailored approach, considering the type of PS and the accompanying treatment conditions, is essential for maximizing the immunogenicity of the cancer cell death induced by PDT. The integration of PDT with other cancer therapies can potentially create a synergistic effect that enhances the overall antigenic yield and the efficacy of the anti-tumor immune response. The assessment of ICD’s quality and quantity revolves around the measurement of key biomarkers: CRT exposure, the pre-apoptotic release of ATP, HMGB1, the presence of type I interferons. The measurement of these biomarkers would be critical for evaluating the efficacy of cancer treatment protocols. ELISA, flow cytometry, and immunohistochemistry are generally used techniques to quantify these markers. In addition, the quantification of specific T-cell responses against tumor antigens can provide more insight into how much immunogenic cell death was induced. To advance the field, further research is needed to establish standardized sensitive assays and thresholds for these biomarkers, which could facilitate the comparison of ICD across different treatment protocols and ultimately guide the selection of the most effective therapeutic regimen.

### 2.2 PDT and TME modulation by affecting stroma, vasculature, and immune cells

PDT has been shown to modulate the TME by affecting various cell types, including immune cells, stromal cells, and vascular endothelial cells. Moreover, PDT can influence the infiltration of different immune cells into the TME, such as macrophages, natural killer cells, and T cells. PDT can also modulate the activity of stromal and vascular endothelial cells, affecting their interaction with immune cells and the extracellular matrix (54, 55). However, tumor hypoxia induced by the destruction of tumor blood vessels and the complexity of the TME with its heterogeneous cell population can limit the effectiveness of PDT-based treatments.

#### 2.2.1 Effects on the stroma

**Figure 2:**
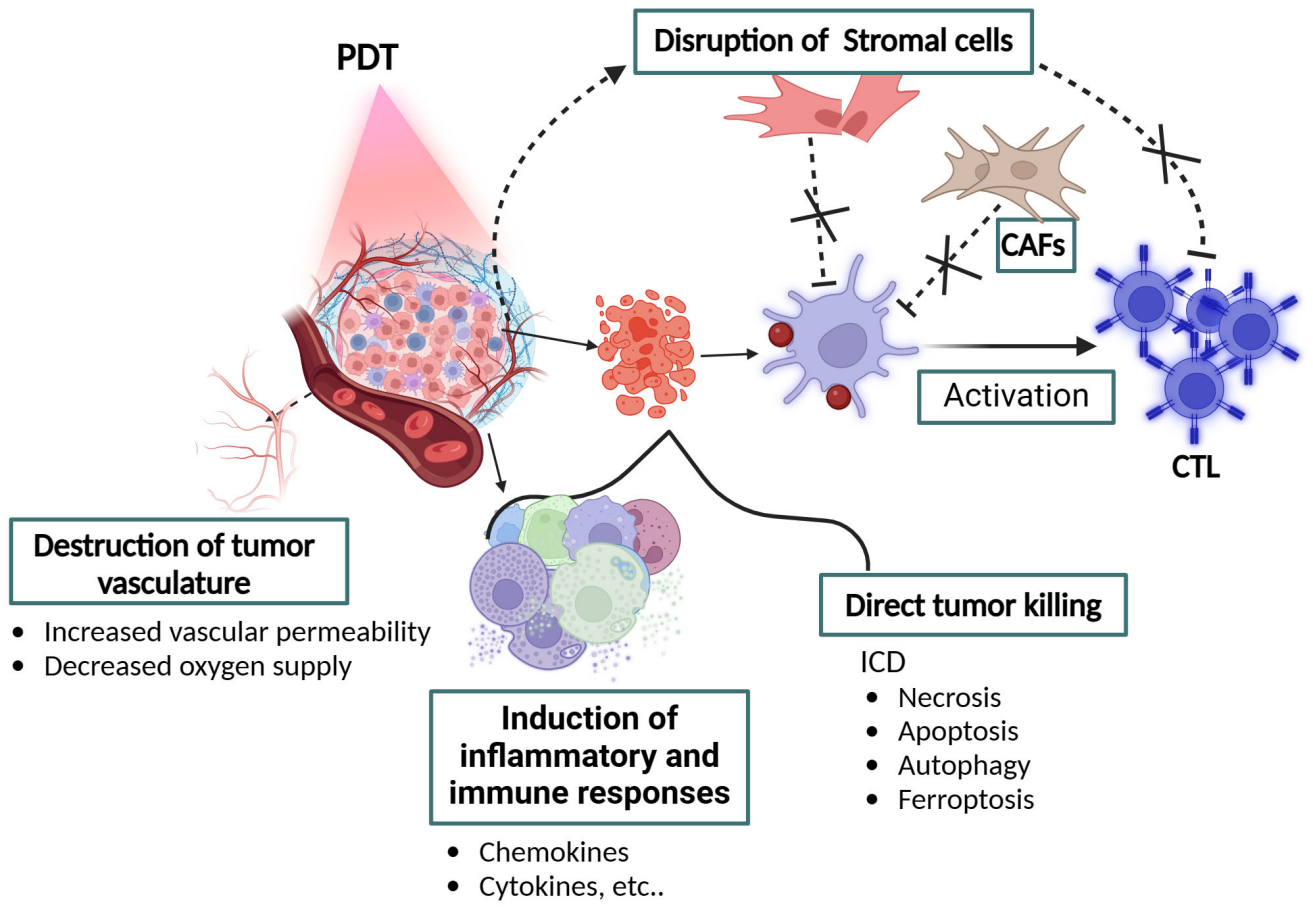
Schematic illustration of PDT-mediated cancer immunity induction mechanism. Three mechanisms underlie the PDT-mediated cancer immunity *in vivo*: (1) direct killing of tumor cells resulting in immunogenic cell death (ICD), (2) damage to the vasculature, and (3) inflammation and immune responses. Figure generated with Bio Render.

Deciphering the complex interactions among components within the TME is crucial for enhancing the efficacy of cancer immunotherapy by combining PDT. The TME is a heterogeneous mix of cancer cells, immune cells, stromal cells, and components of the extracellular matrix, including specialized cells such as cancer associated fibroblasts (CAFs), mesenchymal stem/stromal cells (MSCs), cancer associated adipocytes (CAAs), tumor endothelial cells (TECs), and pericytes (PCs) (54, 55). These cells significantly influence tumor progression and immune evasion, fostering a tumor-supportive environment for the tumor through dynamic interactions (56). PDT, which leverages light-activated PS to generate cytotoxic ROS, targets these supportive stromal cells also resulting in the disruption of tumor-promoting environment within the tumor tissue. This approach not only reduces the tumor burden, but also shifts the TME toward a state less favorable for tumor progression. Recent advancements focus on targeting tumor stromal cells to improve the accuracy and effectiveness of cancer treatment. These strategies aim to (i) thwart stromal cell entry into the TME, (ii) deplete pro-tumorigenic stromal cells, (iii) inhibit stromal cell secretion of growth factors and exosomes, (iv) modify the extracellular matrix composition to inhibit tumor growth (57). Single-cell RNA sequencing (scRNA-seq) offers valuable insights into TME complexity, enabling the identification of distinct stromal cell subtypes. This approach paves the way for developing personalized therapeutic strategies targeting specific subtypes, ultimately enhancing the precision of cancer treatment (56). Despite the potential of these targeted strategies, clinical validations remain in preliminary phases, highlighting the need for further research. A notable advancement in PDT is the development of a nanodrug named MSNs@RA/GA co-loaded with retinoic acid and gallinic acid within mesoporous silica nanoparticles. This formulation specifically targets CAFs, aiming to reverse their pro-tumorigenic state and enhance PDT efficacy. Additionally, the nanodrug modifies the TME, favoring the infiltration of immunostimulatory cells over immunosuppressive ones, showcasing promise in sensitizing tumors to PDT (58). Moreover, recent discoveries emphasize the role of CAF-derived TGFβ1 in inducing resistance to PDT in specific cancers, like cutaneous squamous cell carcinoma. This resistance could be overcome by employing TGFβ1 receptor inhibitors, suggesting the potential of CAF-derived TGFβ1 as a biomarker for PDT responsiveness and for tailoring treatments to individual tumors (59). Integrating PDT with immunotherapy, leveraging its immunostimulatory effects, holds promise for significantly boosting anti-cancer immunity. Studies reveal that low-dose PDT can augment the secretion of pro-angiogenic factors and enhance the immunogenicity of MSCs, underscoring the multifaceted role of PDT in cancer treatment and its potential to redefine therapeutic strategies within the TME (59). As shown in **Figure 2**, the disruption of immunosuppressive stromal cells with PDT will further enhance the therapeutic efficacy of PDT in combination with immunotherapy.

#### 2.2.2 Effects on the vasculature

PDT can directly damage TME vascular endothelial cells and subsequently interfere with the blood supply to cancer cells. This may enhance the infiltration of immune cells into the TME and promote anti-tumor immune responses. However, tumor hypoxia can also induce the expression of immunosuppressive factors such as vascular endothelial growth factor (VEGF) and programmed death ligand 1 (PD-L1), which can limit the effectiveness of the immune response (60, 61). Strategies to overcome these immunosuppressive factors, such as combining PDT with VEGF inhibitors or hypoxia-activated prodrugs, are being explored to improve treatment outcomes. The PS-activated ROS irreversibly destroys tumor cells and nearby endothelial cells. The damage to tumor micro vessels and capillaries leads to acute inflammation (62). During the early stages of PDT, various mechanisms potentiate the detrimental effects of ROS-mediated endothelial damage on the blood supply to the tumor. These include platelet aggregation, edema formation, thrombus formation, thromboxane release, and complement cascade activation. These events lead to further damage to the endothelial cells, followed by vasoconstriction and increased permeability of the blood vessel walls (63). The membrane attack complex generated by complement cascade activation contributes to impaired blood supply in PDT-treated tumors (64). Furthermore, PDT-induced oxidative stress promotes the activation of the complement system and the infiltration of inflammatory cells, which involves the Hsp70-TLR2/TLR4-NFκB axis (65). The vascular endothelial injury caused by PDT results in hypoxia, which leads to elevated levels of hypoxia-inducible factor 1α (HIF-1 α). HIF-1α activation can promote immune escape and immunosuppression by activating TAMs, myelosuppressive cells (MDSCs), lymphocytes, and dendritic cells (DCs) (66). Hypoxic characteristics of solid tumor TME limit PDT therapeutic effects. Moreover, the oxygen consumption in TME following PDT would overload the tumor hypoxia, which will promote tumor growth, metastasis, and invasion, resulting in a poor prognosis of treatment (67). In this context, many efforts have been made to increase the oxygen content in TME to enhance PDT efficacy (68). The rationale is that while PDT-induced insults to the tumor vasculature would have contradictory effects on the therapeutic outcomes: compromised blood supply to TME should accelerate tumor cell death. At the same time, HIF-1α-mediated hypoxic response would result in tumor-promoting consequences.

#### 2.2.3 Effects on immune cell population

PDT significantly impacts the immune system, changing immune cell populations and functions. It destroys tumors and prompts an acute inflammatory response by generating ROS, which mobilizes dendritic cells (DCs) and macrophages. These cells trigger subsequent innate and adaptive immune responses, including memory formation (69). A notable immediate effect of PDT is the surge in neutrophils, attributed to Tumor necrosis factor α (TNFα) induced by ROS, marking the beginning of the immune system’s assault on the tumor (70). Macrophages, essential for the immune-mediated effects of PDT, proliferate and become selectively activated in a dose-dependent manner with low-dose PDT. They release compounds that enhance their tumor-fighting capabilities, including lysophosphatidylcholine which is a precursor of the macrophage-activating factor (70). Additionally, PDT bolsters macrophage phagocytic activity, aiding the clearance of necrotic cells. The amplified immune response involves CD4^+^ and CD8^+^ T cells, which deliver cytotoxic actions against the tumor, signifying PDT’s role in inducing tumor specific adaptive immunity (71). The ROS produced during PDT not only target cancer cells but also stimulate a comprehensive immune response, enhancing both phagocytic activities and B cell-mediated antibody production (72). PDT’s effectiveness and the nature of elicited immune response are influenced by several factors, including the type of cell death induced (immunogenic or non-immunogenic), the light doses used, and the overall therapeutic protocol. Unlike traditional cancer treatments that mainly induce apoptosis, PDT’s ability to trigger necrosis and ICD leads to a controlled inflammation and a specific immune response (73–76). This response is facilitated by the secretion of cytokines and immune mediators during PDT, further involving acute phase proteins and the complement system (64, 77–79). PDT significantly reshapes the TME to activate broad innate immune defenses, such as igniting phagocytic cells and stimulating the release of chemokines and cytokines, thereby inducing a strong inflammatory response (80). Beyond these general defenses, PDT specifically triggers adaptive immune responses through potentiated antigen presentation and selective expansion of lymphocytes, equipping them for the enduring recognition of tumor-specific antigens (73, 74, 81). This capability underscores the dual impact of PDT: not only does it combat primary tumors through ICD, but it also contributes to a sustained anti-tumor effect through induction of memory immune responses (82, 83). This effect is influenced by several factors, including the tumor’s inherent immunogenic properties, the specifics of the PDT protocol, and the patient’s preexisting immune status (84). PDT-modulated cytokines play a pivotal role here, with pro-inflammatory cytokines enhancing immune cell proliferation and anti-inflammatory ones dampening responses (85–88). PDT’s influence extends to cytokine modulation, as demonstrated by recent studies showing changes in VEGF, CXCL9, HIF-1α, and PD-L1 levels post-therapy (89–91). These alterations in cytokine levels not only signal a dynamic local inflammatory response but also influence the balance between pro-inflammatory, which promote immune cell proliferation, and anti-inflammatory activities, which might temper immune reactions. Understanding the fluctuations in these cytokine levels before, during, and after PDT can offer critical insights, guiding the optimization of treatment protocols.

### 2.3 Prospects concerning the synergistic cooperation between immunotherapy and PDT

The past decade has witnessed that immunotherapy has become a dominant pillar in treating cancers. This development has been largely motivated by a deeper understanding of how cancer cells evade immunological surveillance and become resistant to conventional therapies. The discovery of ICD also contributed to the development of more intelligent combinatorial immunotherapy strategies against a wide spectrum of cancers. ICI therapy took its central position in combinatorial cancer therapies. Almost every type of cancer has been tried by ICI therapy using diverse agents from multiple manufacturers. To the despair of people fascinated with the earlier dramatic successes, ICI therapies show durable therapeutic effects only in 20-40% of patients. It became evident that complex immunosuppressive TME should have compromised the ICI activities. As discussed above, PDT could be a safer option for physical TME modulation than RT while the two physiotherapeutic modalities share ICD-inducing characteristics. Long before the ICD concept came out, PDT had been proven to induce anti-tumor immunity as early as 1994 (92).

#### 2.3.1 PDT-generated cancer vaccines

The first efforts used lysates/supernatants from Photofrin-PDT treated cells (PDT-based lysates vaccine), which was tested as a prophylactic vaccine in a mouse mammary tumor model. The vaccine showed significant tumor growth suppression. Such an anti-tumor effect was tumor-specific and correlated with DC maturation and IL-12 production. Importantly, in the same experimental system, no protection was observed for vaccines prepared by ionizing radiation, UV light, hyperthermia, or freeze/thaw cell lysis (37). This result strongly suggests that ICD was induced only by PDT and immunogenic tumor antigens were liberated from PDT-affected cancer cells. Later, similar results were reported with other PSs such as benzo-porphyrin derivative, chlorin, hypocretins, hematoporphyrin, and redaporfin, with strong immunologic evidence showing the involvement of lymphocytes and dendritic cells (34, 93–95). Based on the successful prophylactic vaccine results, therapeutic vaccine strategies were tried using pre-established tumor models with substantial success (96). These studies revealed that PDT-based lysate or whole-cell vaccines are tumor-specific, which means that protection is limited to rechallenges with the same cell types (96). PDT-generated whole-cell vaccines elicited an accumulation of DCs and their functional maturation (37). Maturation of DCs and interferon-producing T cells are also often reported, as well as the loss of effect when immunodeficient mice were used (96). Immature DCs co-incubated with vaccine cells undergo phenotypic maturation and become IL-12 producers (96). As PDT-generated lysate whole-cell tumor vaccines require the crucial role of DCs to induce antigen-specific cytotoxic T-cell responses, many PDT/immunology research groups later adopted the DC vaccine strategy. Optimally activated DCs induce enhanced immunological memory, contributing to the effective suppression of recurrence and metastasis. Dying/dead cancer cells by PDT should provide excellent tumor antigens and DAMPs generated by ICD. For example, EMT6 mammary cancer cells killed with PDT were pulsed to immature DCs. The matured tumor antigen-pulsed DC vaccine exerted a significant tumor suppression correlated with the generation of antigen-specific IFNγ-producing lymphocytes in the spleen. In contrast, notable tumor suppression was not observed in animals vaccinated with DCs pulsed with freeze/thaw-killed tumor cells or PDT-treated tumor lysates (97).

ALA (aminolaevulinic acid)-PDT was also used to induce ICD in the skin squamous carcinoma PECA cell line, which was pulsed to immature DC cells. ALA-PDT-based DC vaccines provided protection to mice against the rechallenge with live cancer cells, while such protection was not observed with freeze/thaw-based DC vaccines (98). DCs pulsed with apoptotic hypericin-PDT-treated Lewis lung carcinoma (LLC) cells also activated DCs, being able to stimulate CD8^+^ T cells to produce IFNγ. PDT-treated cancer cell pulsed DC vaccine showed superior tumor suppression to PDT-treated whole-cell vaccine, which was correlated with increased IFNγ-producing CD8^+^ T cells and decreased Tregs (99). In a peritoneal mesothelioma model, photosensitizer, an OR141-PDT-based DC vaccine, was administered intraperitoneally. The PDT-based DC vaccine-treated group showed significantly extended survival compared to the group treated with anti-CTLA4 antibody. Enhanced CD8^+^ and CD4^+^ T cell response was noted both locally and systemically, with strong IFNγ^+^ T cell infiltration in mesothelioma tumors. The DCs pulsed with PDT-killed mesothelioma cells also manifested significantly increased expression of CCR7, suggesting more efficient homing to T cell areas of draining lymph nodes (100).

High-grade malignant glioma and glioblastoma (GBM) are aggressive types of primary brain tumors that are almost universally fatal despite some progress in treatment and management. Malignant brain tumors generally form cold (immunosuppressive) TME, which should account for the unsuccessful ICB treatment outcomes (101). Unfortunately, all immunotherapies tested to date have failed to improve clinical outcomes in unselected cohorts of patients with GBM (102). In a recent meta-analysis, the active immunotherapy of GBM reduced the risk of 2-year mortality by as much as 2.5% compared to the control group (103). Cancer cells undergoing ICD by appropriate PDT should potentially activate autologous DCs by providing highly immunogenic tumor antigens and strong DAMP signals. A preclinical study proved this possibility well (104). The study used a hypericin-PDT-treated mouse glioma cell line (GL261), being partially immunotherapy-susceptible, and demonstrated that these dying cells could efficiently induce the maturation of DC. Vaccination experiments revealed that the hypericin-PDT-based DC vaccine induced a strong anti-tumor immune response, efficaciously protecting mice from intra-brain challenge with homologous live cancer cells. The ability of DC vaccines to elicit tumor rejection was significantly blunted if cancer cell–associated reactive oxygen species and emanating danger signals (CRT, ATP, HMGB1) were blocked singly or concomitantly. In a curative setting, hypericin-PDT-based DC vaccines synergized with standard-of-care chemotherapy (temozolomide) to increase the survival of high-grade glioma-bearing mice by ~300%, resulting in ~50% long-term survivors. The DC vaccine induced an immunostimulatory shift in the brain TME from regulatory T cells to Th1/cytotoxic T lymphocyte/Th17 cells (53).

#### 2.3.2 PDT in combination with immunotherapeutic modalities: ICI and other immunomodulators

##### 2.3.2.1 ICI’s gleam and gloom: hopes with PDT

**Figure 3:**
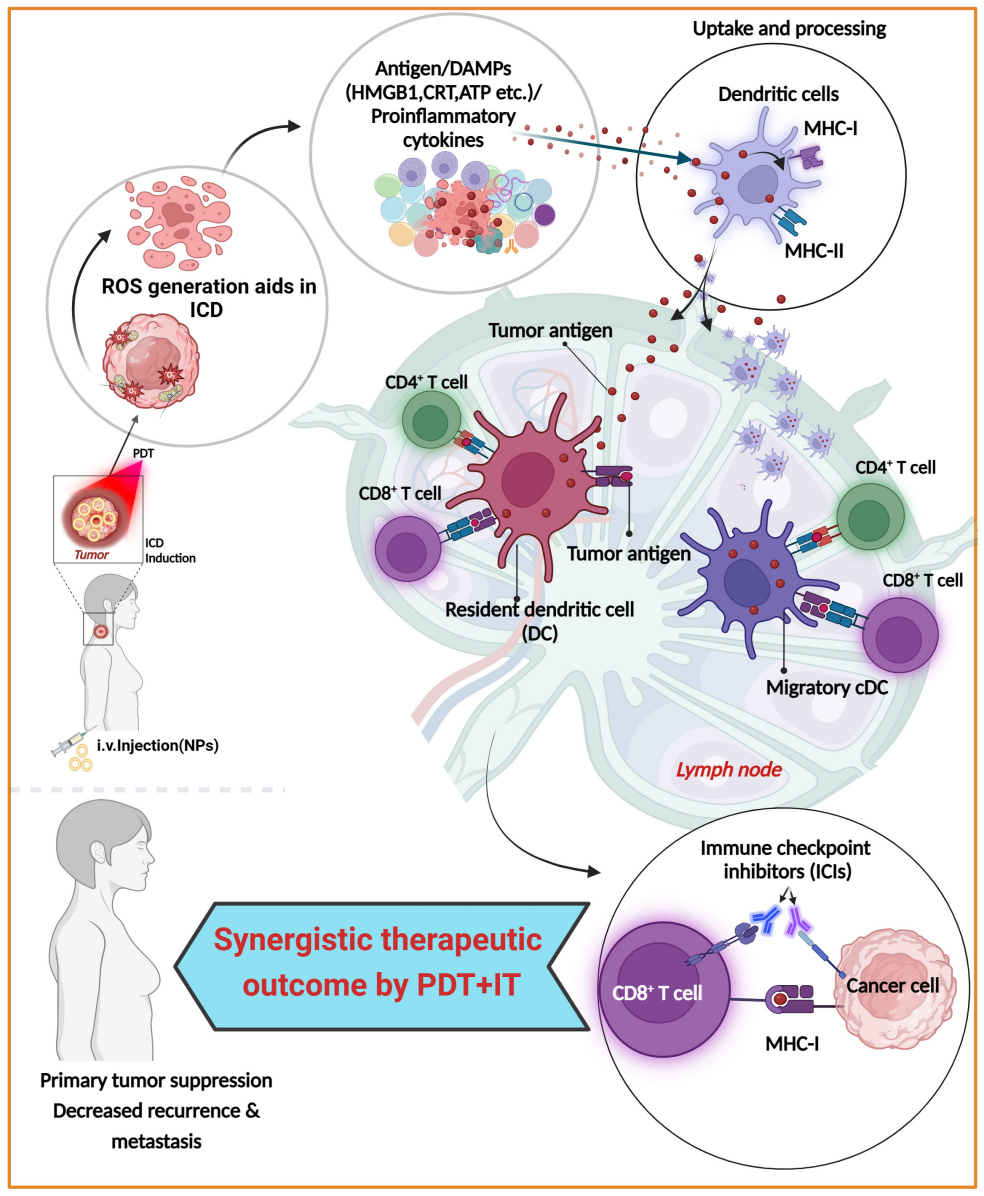
An overview of combinatorial cancer treatment employing photodynamic and immunotherapy. Immunogenic antigens and DAMPs released from dying tumor cells would activate APCs, which will subsequently result in the activation of tumor specific CD4^+^ and CD8^+^ T cells in the secondary lymphoid organs such as LNs and spleen. Activated migratory DCs from tumor sites and resident DCs in LNs activated by tumor antigens translocated through lymphatics play essential roles in activating effector T cells. Checkpoint inhibitors can further invigorate activated and precursor of exhausted T cells to synergistically enhance PDT efficacy. Figure generated with Bio Render.

Monoclonal antibody drugs modulating immune checkpoints have drastically changed the cancer therapy landscape. Given the PDT’s innate effectiveness of physical TME modulation and the ability to induce ICD, it will be natural to expect a synergistic interaction between ICI and PDT. Strategic combinatorial treatment employing ICIs with other therapies, including PDT, would expand therapeutic efficacy. RT could be considered an effective TME-modulating partner for ICI therapeutics. PDT may have the advantage over RT in many aspects as discussed above. PDT’s cytotoxic activities are spatially limited by less tissue-destroying light activation process, limiting PDT’s direct effects on the treatment focus. As **Figure 3** depicts, the photodynamic therapy (PDT) directly kills cancer cells by enhancing the ROS and releases tumor-specific antigens for T cell activation. In concert, ICIs enhance T cell-mediated tumor cell killing by blocking immunosuppressive interactions, which will result in potentiated tumor growth suppression and spread reduction.

When activated through the T-cell receptor (TCR) and CD28 signaling, T cells proliferate and produce cytokines, while the expression of inhibitory molecules (such as PD-1) is triggered at the same time. In other words, T cell priming elicits an activation program and a parallel program that will eventually attenuate the response. Therefore, T cells have limited time to be aggressive before they are reined, not to damage normal bystander cells (105). When the tumor grows, TME exploits the reining phase of the T cell responses. When combining ICIs with ICD-inducing/TME-modulating PDT, the choice of ICI should be based upon scientific speculation about which phase of T cell status and what type of immune cells should be targeted. CTLA4 is expressed immediately following engagement of TCR, with peak expression around 48 to 72 hours following T-cell activation. It competes with CD28 in binding to B7-1 (CD80) and B7-2 (CD86) molecules on APCs with higher affinity than CD28, leading to inhibition of costimulatory CD28 signaling, thereby dampening T-cell signaling (106). Anti-CTLA4 primarily functions in T-cell priming and expands clonal diversity, which is not observed with anti-PD-1/PD-L1 treatments. Anti-CTLA4 predominantly affects CD4^+^ T cells, with an increased Th1 subset of CD4^+^ T cells expressing ICOS (105). Anti-CTLA4 can also promote T-cell trafficking into immunologically “cold” tumors (105).

The T cell PD-1 expression is noted within 24 hours after TCR engagement (107). PD-1 expression is maintained by γ-chain cytokine stimulation (e.g., IL-2, 7, 15, and 21) and transcription factors (FOXO1 and NFAT) (108, 109). Its ligands PD-L1 (B7-H1, CD274) and PD-L2 (B7-DC, CD273) are expressed in a variety of cell types—leukocytes, nonhematopoietic cells, and cancer cells (109). Type 1 and type 2 interferon signaling (particularly IFNγ), as well as other inflammatory cytokines, including GM-CSF, IL-4, IL-10, IL-17, IL-27, and TNF-α, are strong inducers of PD-L1 and PD-L2 (109). Any anti-cancer therapeutic regimen triggering host proinflammatory responses would induce PD-L1 expression in residual tumor tissue on cancer and bystander cells, which will interfere with further tumor suppression by activated host immune responses. Moreover, MDSCs in TME induce PD-L1 expression in tumor cells through an EGFR/MAPK-dependent mechanism (110). Anti–PD-1/PD-L1 antibodies predominantly affect exhausted CD8^+^ T cells. Unlike anti-CTLA4, anti–PD-1/PD-L1 therapeutics do not expand clonal diversity nor promote T-cell trafficking into “cold” TME (105).

Because of their non-overlapping action mechanisms, concurrent targeting of CTLA4 and PD-1/PD-L1 can improve therapeutic efficacy than each monotherapy (105). In the phase III CheckMate 067 trial (NCT01844505) in advance melanoma, durable clinical benefit was demonstrated with nivolumab (anti-PD-1) plus ipilimumab (anti-CTLA4) combination over monotherapy with nivolumab or ipilimumab (111). Though the combination of anti-PD-1 and anti-CTLA4 brought notable survival improvement in *BRAF*-mutant patients, a marginal effect was observed over anti-PD-1 treatment. In the CheckMate 227 (NCT02477826) Part 1 trial, OS of patients with metastatic non-small-cell lung cancer treated with nivolumab plus ipilimumab was compared with chemotherapy regardless of tumor programmed death ligand 1 (PD-L1) expression status. At a minimum follow-up of 61.3 months, 5-year OS rates were 24% versus 14% for nivolumab plus ipilimumab versus chemotherapy (PD-L1 ≥ 1%) and 19% versus 7% (PD-L1 < 1%) (112). Recently finished CheckMate 651 trial (NCT02741570), which evaluated first-line nivolumab plus ipilimumab versus EXTREME (cetuximab plus cisplatin/carboplatin plus fluorouracil ≤ six cycles, then cetuximab maintenance) in recurrent/metastatic squamous cell carcinoma of the head and neck showed no superiority of the ICI combination over the antibody and chemotherapy combination regimen (113). These phase III clinical trial results show that ICI combination improved survival of patients compared with conventional therapies in responsive tumors, while therapeutic superiority was not proved in refractory tumors. Melanoma, non-small-cell lung cancer, and head and neck squamous cell cancer would be well treated with physiotherapies such as RT and PDT in combination with ICIs. On the other hand, some tumors that are resistant to PDT present a therapeutic hurdle by upregulating the expression of innate or adaptive immune checkpoints, which provide favorable conditions for tumor progression in pre-existing TME. With this background, combinations of ICIs with PDT were actively explored in diverse preclinical cancer models of PDT-refractory tumors, and many positive experimental results have been reported during the last decade (114).

##### 2.3.2.2 Recent progress in IT-PDT combination

The poor response to ICI therapy could be attributed to four main reasons, which could be overcome by appropriate PDT strategy: 1) tumor antigen deficiency, 2) insufficient infiltration of T lymphocytes, 3) defects in the tumor antigen processing and presentation mechanism, and 4) an immunosuppressive TME (115). If ICD is appropriately induced *in vivo* by PDT, all these four reasons could be overcome to let ICIs exert their full functionalities. Numerous reports of preclinical studies combining PDT and antibodies against checkpoints PD-1/PD-L1, CTLA4, CD47, and IDO (indolamine 2,3-dioxygenase) are increasingly appearing in the literature. Those studies use murine experimental models of colorectal, breast, renal, lung, cervical, head, and neck or skin cancers (114, 116). Here, we review more advanced ICI-PDT combination approaches. Shuang Li et al. treated head and neck squamous cell carcinoma with DCs pulsed with SCC7 cells whose ICD was induced by PDT, which was combined with anti-PD-L1 monoclonal antibody. The PDT-DC vaccine and anti-PD-L1 synergistically suppressed tumor progression (117). Another promising approach is preferentially concentrating PS at TME by either passive or active targeting for better safety and combining ICIs to maximize anti-tumor immunity. Kexin Li et al. adsorbed anti-PD-L1 to a cationic liposome encapsulating photosensitizer HMME and administered the nanocarrier formulation intravenously to tumor-bearing mice. After confirming that the nanocarrier preferentially accumulated in tumors, they carried out PDT. Tumor cells underwent ICD, and anti-PD-L1 was released in the TME. The PD-L1McAb&HMME generated profuse ROS to induce ICD *in vivo* and robustly activated immune systems to suppress primary tumor growth and metastasis. In addition, longer-lasting immune memory was noted in the treated animals (118). Active targeting of tumors should be an ideal way of accumulating PS in TME. Tong et al. formulated a cyclic RGD (cRGD)-modified liposomal delivery system loaded with anti-PD-L1 and PS pheophorbide A and tried to target 4T1 mouse breast cancer cells with low PD-L1 expression by recognizing overexpressed surface α_v_β_3_ integrin. The PDT responsively elevated the expression of PD-L1 on the tumor cells. PDT, in combination with the anti-PD-L1 therapy, promoted the activation and maturation of DCs as well as the infiltration of cytotoxic T lymphocytes, resulting in the augmented antitumor immune response for the enhanced therapeutic effect (119). This study suggests that PDT could induce PD-L1 expression in treated tumor cells, and co-administered ICI will help activate T lymphocytes to kill cancer cells more effectively. Kaneko et al. reported a tumor-specific PS (HS201) that binds heat shock protein 90 expressed in cancer cells. The combination of HS201-PDT with anti-PD-L1 antibody demonstrated greater antigen-specific immune response, tumor growth suppression, prolonged mouse survival time, and abscopal effect. The most significant increase of intratumorally activated CD8^+^ T cell infiltration and decreased exhausted CD8^+^ T cell number were observed following the HS201-PDT-anti-PD-L1 combination compared with HS201-PDT monotherapy. Markedly enhanced CXCL, galectin, GITRL, PECAM1, and Notch signaling were noted in the combination group, along with CD8^+^T cell activation in the combination group. The highly enhanced expression of the CXCR3 signature was observed in the combination group, explaining the enhanced tumor infiltration by T cells (120). With the development of a novel theragnostic PS, porphyrin lipoprotein (PLP), Zheng’s lab combined PDT with ICI to enhance abscopal tumor suppression (121). PLP is a ∼20 nm, multimodal biomimetic nanoparticle serving as a PS, fluorophore, and PET imaging agent upon chelation with Cu-64. When PDT was performed on a highly aggressive mesothelioma mouse model four times, termed repeated PDT (R-PDT), irradiated tumors were eradicated, while non-irradiated (abscopal) tumors had a delay in tumor growth. The R-PDT + αPD-1 combination induced broader innate immune activation. There was a greater propensity for antigen presentation in the spleen and distal non-irradiated tumor-draining lymph nodes, where DCs and macrophages had increased expression of MHC class II, CD80, and CD86. Concurrently, there was a shift in the proportions of CD4^+^ T cell subsets in the spleen and an increase in the frequency of CD8 T cells in the non-irradiated tumor draining lymph nodes. Histology revealed an absence of gross inflammation in critical organs after the combination of R-PDT + αPD-1. They have emphasized that the abscopal effect was enhanced by the combination αPD-1 with R-PDT with minimal toxicities (61).

Nanocarriers are designed to deliver PS and ICI to TME with higher efficiency by passive targeting exploiting tumor vasculature structure or active targeting using TME-specific ligands. To deliver PDT at the right place and timing, the nanocarriers were engineered to be traced by imaging and activated by photo radiation after confirming the preferential accumulation at targeted TME (122, 123). ICI was administered following targeted PDT synergistically enhanced tumor suppression. Chen et al. reported an activatable strategy employing a cleavable linker by PDT activation (124). They developed a photodynamic immunomodulator, ICy-NLG, by conjugating the photosensitizer ICy-NH_2_ with the indoleamine 2,3-dioxygenase one inhibitor NLG919 through a glutathione (GSH)-cleavable linker to achieve activatable PDT. Though the conjugation considerably suppressed both the PDT effect and the activity of the inhibitor, the PDT effect was restored and led to the ICD of tumor cells after ICy-NLG was activated by high levels of GSH in TME. The released tumor-associated antigens, in conjunction with the activated immune checkpoint inhibitor, induced a synergistic antitumor immune response, suppressing primary and distant tumors and preventing lung metastasis. Zheng et al. designed a self-delivery biomedicine (CeNB) based on PS chlorine e6 (Ce6), IDO inhibitor (NLG919), and PD1/PDL1 blocker (BMS-1). CeNB carried fairly high drug content (nearly 100%), favorable stability, and uniform morphology. CeNB-mediated cascade IDO and PD1/PDL1 inhibition robustly modulated immunosuppressive TME toward immune activation. The PDT of CeNB not only inhibited tumor proliferation but also induced ICD response to activate cascade immune responses. Ultimately, self-delivery CeNB tremendously suppressed the tumor growth and metastasis with minimal side effects (125). The PS/ICI combinatorial therapy studies were summarized in **Table 1**.

**Table 1:** Summary of recent work on the application of PDT and Immunotherapy activating immune response.

Material	Photosensitizers	Activation method	Combined immunotherapy	Nature of immunotherapeutics	Cancer model	Outcome	Advantages	Ref
**UCNPs**	ICG & RB	Cancer vaccine (protein antigen)	TSA & anti-CTLA-4	maleimide captured TSA	4T1 allografts	Achieved long term survival in 84% of mice, with 34% producing tumorspecific immunity	Enhanced efficacy	(119)
**BONVs**	BPQD	Immune adjuvant	CpG ODNs	BONVs were transformed into BPQD and released CpG under NIR laser radiation	4T1 allografts, MCF-7 (human breast cancer) xenografts	Significant inhibition of distant tumors and lung metastasis	Effective drug delivery & excellent biocompatibility	(126)
**nMOF-CPG**	nMOF	Immune adjuvant	CpG ODNs & antiPD-L1	Combined formulation of antigen and anti-PD-L1	TuBO (mouse breast cancer) tumor allografts	Over 97% tumor regression.	Streamlined synthesis & high efficacy	(127)
**PC2GCpD** **(Gd)**	IR820	Immune adjuvant	CpG ODNs	Direct administration of immunomodulatory agent with photothermal nano agents.	EMT6 allografts, RAW 264.7 (Mouse monocyte macrophages). NIH3T3 (mouse embryonic fibroblast)	Tumor growth was almost completely inhibited	Optimal biosafety & advanced imaging potential	(128)
**PTX-R837-** **IR820@TSl**	IR820	Immune adjuvant	R837 & Pa	The ROS and heat generated by IR820 triggered the release of the therapeutic agent	MFC (mouse gastric cancer allografts)	Treatment resulted in a 100% survival	Durable efficacy & excellent bioavailability	(129)
**M/C/AN**	Ce6	Immune adjuvant	MPLA	To enhance therapeutic effect, immune adjuvant was delivered together with the nanoparticle.	B16F10 (mouse melanoma allografts)	All mice survived beyond 40 days post treatment	Simplified synthesis & enhanced efficacy	(130)
**CCPS/HPPH/DOX**	HPPH	Immune adjuvant	DOX & CCPS	CCPS with amine groups used as an adjuvant carrier for photo immunotherapy	MC38 (mouse colon cancer)	50% survival at 36 days post-inoculation	Versatility of nano formulations & enhanced efficacy	(131)
**UCNP-Ce6R837 NPs**	UCNP	ICI	Anti-CTLA4 & R837	Multifaceted approach targeting CTLA-4 and TLR7	CT26 (mouse colon adenocarcinoma allografts)	Marked inhibition of primary and distant tumor growth, tumor recurrence was prevented	Improved tumor penetration & efficacy	(132)
**FeS-** **Gox@PTX**	FeS nanodot	ICI	Anti-CTLA4 & PTX	FeS-Gox@PTX – based nano-delivery system can penetrate tumor deeply and realize starvation therapy	4T1(mouse breast cancer allografts)	Substantial reduction in primary tumors and distant metastases	Enhanced tumor targeting, multifaceted approach	(133)
**NCP@pyro**	Pyro lipid	ICI	Anti-PD-L1 & oxaliplatin	Chemo- photoimmunotherapy with nanoscale coordination polymers	HT29 xenograft, CT26 and MC38 allograft	Significant inhibition of primary and metastatic tumor growth	Optimized drug delivery, integrated therapeutic strategy	(134)
**ZnP@pyro**	Pyro lipid	ICI	Anti-PD-L1	PDT based on ZnP@pyro with extremely high biocompatibility makes tumor sensitive to ICI through chem-PDT	4T1 and TUBO allografts	The primary tumor was eliminated, and the decreased volume in distal tumors	Biocompatibility, efficacy	(135)
**Micellar complex**	Pheophorbide A	ICI	PD-L1 siRNA	The pH responsive micellar complex encapsulated PDL1 siRNA and photosensitizers in the same micelle to target the same cancer cell	B16F10 xenografts	Tumor eradication achieved with enhanced survival, reaching 75% in mice	Efficacy, acid responsive drug release	(136)
**Nano micelle**	MTPP	ICI	PD-L1 siRNA	A pH-responsive nano micellar is loaded with a mitochondrial targeting photosensitizer and PD-L1 siRNA	B16F10 xenografts, 4T1 xenografts	Tumor reappearance rates diminished to 25%	Mitochondrial targeting, acid responsive drug release	(137)

Anti-CTLA-4, Antibody against CTLA-4 (Cytotoxic T-lymphocyte-associated protein 4); Anti-PDL1, Antibody against PD-L1 (Programmed death-ligand 1); BONVs, Bismuth Oxychloride Nano vehicles; CCPS/HPPH/DOX, Cyclic CPS (Polymersome)/HPPH (2-devinyl-2-(1-hexyloxyethyl)py/(Doxorubicin); Ce6, Chlorin e6, a photosensitizer; CpG ONDs, CpG Oligodeoxynucleotides; FeS nanodot, Iron sulfide nanodot; FeS-Gox@PTX, Iron Sulfide Nanoparticles conjugated with Glucose oxidase (Gox) and Paclitaxel (PTX); ICG, Indocyanine green; IR820, Imiquimod, an immune response modifier; M/C/AN, Hydrophobic chlorin e6 (C)and monophosphoryl lipid A (M) amphiphilic phenylalanine (AN); MPLA, Monophosphoryl Lipid A, an immune stimulant; MTPP, (5-(3Hydroxy-p-(4-trimethylammonium)butoxyphenyl)-10, 15, 20-triphenylporphyrin chlorine; NCP@pyro, Nanoscale Coordination Polymers with pyrophoric properties; nMOF, Nanoscale metal-organic framework; oxaliplatin, A platinum-based chemotherapy drug; PC2GCpD(Gd), ODN, Oligodeoxynucleotide; Versatile nano assemblies; siRNA, Short interfering RNA; PTX, Paclitaxel; R837, Imiquimod, an immune response modifier; RB, Rose Bengal; TSA, Tumour specific antigen; UCNP, Up conversion nanoparticles;UCNP-Ce6-R837 NPs, Up conversion Nanoparticles conjugated with Chlorin e6 (Ce6) & particles Imiquimod (R837); ZnP@pyro, Zinc pyrophosphate nanoparticle that encapsulated pyropheophorbide-lipid conjugate.

Meanwhile, the exploration of peptide cancer vaccines, specifically those tailored to target personalized neoantigens, is a vibrant and dynamic field of research (138). Our group is also getting interested in TME targeting and enhancing PDT-ICI combination cancer therapy. We have combined the cancer peptide antigen vaccine with PDT-ICI treatment. We combined PDT agent Pheophorbide A along with FlaB-Vax (a flagellin-adjuvanted tumor-specific peptide) and investigated its promotion of PD-1 blockade-mediated melanoma suppression using an abscopal mouse B16-F10 melanoma cancer model. The systemic antitumor immune responses for local and abscopal tumor control, significantly increased by increasing the tumor-infiltrating effector memory CD8^+^ T cells and systemic IFNγ secretion along with the accumulation of migratory CXCL10-secreting CD103^+^ DCs, contributing to tumor antigen cross-presentation in the TME (31). Flagellin serves as an efficacious adjuvant for peptide cancer vaccine (139–143), and is a resilient protein that maintains integrity under harsh physical conditions such as high temperature, acidity, and ROS (144). In this regard, flagellin would also serve as an excellent *in situ* vaccine adjuvant for ICD-inducing therapeutic modalities such as RT, PDT, and photothermal therapy.

##### 2.3.2.3 PDT combined with other immunomodulatory agents

When PDT is coupled with immunostimulatory drugs, both local and systemic immunologic responses have been consistently observed across various animal models, resulting in sustained immune activation, increased tumor cell destruction, and reduced tumor growth. Notably, the benefits of such combinations include improved antigen presentation, heightened T cell activation, reduced Tregs, and bolstered resistance to tumor recurrence, irrespective of PS used. Xia et al. illustrated that combining PDT with CpG oligodeoxynucleotide can extend survival, mitigate metastasis, and amplify CD8+ T cell activation. Similarly, another innovative approach by Shams et al. involves a two-stage therapy that integrates a low, immunogenic dose of PDT followed by a higher dose aimed at direct tumor control, resulting in prolonged survival and reduced metastatic growth, albeit with varying results across different tumor cell lines (75). A dual-photosensitizer strategy employing HPPH and Photofrin has also been suggested, where an initial low-dose PDT for immune stimulation is followed by a high-dose treatment for tumor eradication, leading to increased tumor-specific CD8^+^ T cell activation and reduced metastasis in CT26 colon and 4T1 breast carcinoma models (145). When PDT cell lysate vaccines, prepared from radicicin-treated TC-1 cells expressing the human papillomavirus E7 antigen, were combined with the immunoadjuvant CpG, there was a marked suppression of tumor growth. This effect was observed in both preventive and therapeutic settings, indicating a notable enhancement in the immune response, particularly through increased interferon production and CD8^+^ T cell activity, surpassing the outcomes of individual treatments (146). Additionally, a study on a rat tumor model that received radicicin-based PDT along with interleukin-12 delivered by adenovirus (AdmIL-12) showed significant boosts in interferon and TNF production as well as CD8^+^ T cell proliferation. This led to the complete regression of established tumors in the mice, highlighting the potential for radicicin to enhance the efficacy of PDT combined with immunotherapy (147). Another investigation utilized fontanin-based PDT in tandem with synthetic long peptides containing tumor antigenic epitopes to target an aggressive T cell lymphoma in a mouse model. This approach successfully elicited a strong antitumor CD8^+^ T cell response, underscoring the potential of combining PDT with targeted immunotherapy strategies to stimulate potent antitumor immune reactions (70). These findings collectively underscore the importance of leveraging a multifaceted approach to cancer therapy that integrates PDT with immunotherapy and other anticancer strategies. The goal is to harness and amplify the immune system’s natural capacity to fight cancer, emphasizing the crucial role of immune system activation in combating cancer.

#### 2.3.3 Photodynamic therapy and nanotechnology provide amenable alternatives to RT

Enhancing the efficacy of PDT through nanoparticle platforms is a promising strategy in cancer therapy. The limited functionality of tumor-infiltrating DCs in presenting tumor-specific antigens is a critical barrier to generating effective antitumor immune responses (148). To address this, the integration of nanoparticle technology in cancer immunotherapeutic regimens has been attempted to bolster antitumor immunity by facilitating the presentation of antigens, which should be the rate-limiting step in initiating anti-tumor immune responses (149). Innovations such as the use of CpG oligodeoxynucleotides, which act as adjuvants by targeting TLR9, have shown promise in clinical studies for enhancing immunity against solid tumors (150, 151). For example, Im et al. developed a mesoporous silica nanocarrier that responds to hypoxia (termed CAGE), which was encapsulated with both Ce6 and CpG adjuvant (150). Once the CAGE accumulates in hypoxic tumor region, azo linkers were cleaved to rapidly release CpG and ROS-induced DAMPs were released from melanoma by local Near infrared (NIR) irradiation. Simultaneously, CpG released earlier recruited DCs and promoted the maturation of DCs leading large number of CTL infiltration (150). Yang et al. on the other hand, developed a polymersome encapsulating HPPH(2-(1-hexyloxyethyl)-2-devinyl pyropheophorbide-a, a chlorin based PS) and doxorubicin (152). The polymersome (CCPS) acts as an adjuvant by its own due to the presence of primary and tertiary amines on the surface (152). The vaccine composed of CCPS/HPPH/Dox polymerosome and subjected to NIR irradiation led to an increase in the presence of mature DCs in lymph nodes and killer T cells at the tumor tissue (152). This was further enhanced by integrating ICD with an adjuvant, which, when combined with PDT, significantly inhibited the growth of both primary and metastatic tumors in experiments conducted on a colon adenocarcinoma cell line (MC38) tumor-bearing C57BL/6 mice (152). In a related study, Cheng and colleagues engineered a unique chimeric peptide, PpIX-PEG8-KVPRNQDWL, which self-assembles into nanoparticles (PPMA) aimed at melanoma targets. This peptide, designed to recognize a specific melanoma antigen (KVPRNQDWL), has been shown to facilitate the entry of cytotoxic T cells into the TME, thereby boosting the efficacy of PDT against malignant melanoma (153). In an effort to enhance the presentation of antigens, Xu and their team created a biodegradable mesoporous silica nanoparticle system that carries both CpG and Ce6. This comprehensive nano system, when evaluated against solo applications of PDT, vaccine, or adjuvant, attracted a higher number of tumor-infiltrating cytotoxic T cells and showed superior anti-tumor performance on both primary and abscopal site MC-38 tumors (154). Hence, this approach of using multiple stimuli has shown to be a highly promising strategy for inducing a strong anti-tumor immune response (154). Recent studies showed that DSPE-PEG-maleimide could form thioether bonds with proteins, this feature of the material can be used to capture tumor-derived antigens from the TME (155). Based on this report, antigen-capturing platforms have been developed for photoimmunotherapy, designed to improve antigen uptake and presentation efficiency (156). These platforms utilize nanoparticles to capture tumor-derived protein antigens during PDT, enhancing the ICD effect and inducing stronger immune responses. This strategy represents a novel approach to increase the specificity and efficiency of anti-tumor immune responses through targeted delivery and presentation of tumor antigens. Liu et al., designed up-conversion nanoparticles (UCNPs) modified with DSPE-PEG-mal., co-loaded with ICG and rose Bengal (RB) as PS. This NIR-triggered antigen-capturing nanoplatform generated significant ^1^O_2_. These nanoparticles also captured tumor-derived protein antigens from dying cells, which could increase the efficiency of antigen presentation to induce a stronger immune response (157).

Binbin Ding (158) created an innovative type of nanoparticle named UCMS, which is a large-pore mesoporous silica-coated up-conversion nanoparticle, notable for its sub-100 nm dimensions and its ability to load significant quantities of biomolecules thanks to its extensive mesoporous framework. This UCMS, when loaded with the PS merocyanine 540 (MC540) and the model protein chicken ovalbumin (OVA), demonstrated enhanced immunostimulatory effects under NIR laser irradiation. Furthermore, when tumor cell fragments were loaded, the combination of UCMS-MC540-TF significantly inhibited tumor progression and improved survival in mice bearing CT26 tumors. In a related development, Huaji Wang encapsulated OVA within nanoparticles using a disulfide bond network among OVA molecules (159). These nanoparticles were then coated with a membrane from B16-OVA cells and incorporated both OVA and the PS Ce6, resulting in the creation of a membrane-cloaked OVA nanoparticle (MON). This MON was shown to trigger the activation of bone marrow-derived dendritic cells (BMDCs) and promote antigen cross-presentation, effectively triggering an immune response *in vivo*. Utilizing the B16-OVA tumor model, MON-facilitated PDT succeeded in completely eliminating primary tumors and establishing a durable antitumor immune memory. Neoantigens, which are unique to each tumor, have emerged as powerful triggers of the immune system’s response against tumors. The evolution of high-throughput omics technologies and neoantigen prediction methods have ushered in neoantigen-based therapies as a significant area of research.

##### 2.3.3.1 Ideal patient population for PDT-based immunotherapy and biomarkers for selection

Generally, PDT based immunotherapy is a versatile treatment modality suitable for a broad patient demographic, notably due to its non-reliance on specific genetic predisposition or tolerance profile (57). Ideal candidates for PDT include individuals with superficial or easily accessible tumors via endoscopy, such as those found in the skin, head and neck, esophagus, or bladder. This preference is attributed to the necessity of direct light exposure for PS activation. Tumors that remain localized and have minimal metastasis are more responsive to PDT, benefiting from precise light direction to the tumor site (160). The success of PDT-based immunotherapy significantly hinges on a patient’s immune system functionality. Individuals with minimal prior exposure to chemotherapy or radiation, which could dampen immune responsiveness, generally exhibit better outcomes with PDT (75). The TME also plays a crucial role, where tumors situated in less immunosuppressive settings—indicated by lower Tregs and MDSCs—are more receptive to PDT based immunotherapy (161). Patients undergoing neoadjuvant therapies could also get benefits from PDT, either through tumor mass reduction pre-surgery or by enhancing tumor antigenicity, thereby augmenting the effectiveness of subsequent immunotherapies (76). Those with tumors resistant to ICIs might be advantaged by PDT due to its capacity to induce ICD, which will release quality tumor antigens and promote DC activation and T-cell priming (162).

Patients whose tumors exhibit specific receptors or antigens suitable for targeting by designated PS would gain a better outcome from PDT. By employing targeted PS, treatments can achieve better tumor eradication while sparing healthy tissues (163). The identification of patients who are likely to respond well to PDT-based immunotherapy hinges on the detection of biomarkers predictive of the therapy success. These biomarkers encompass a range of indicators, including tumor-specific markers, signs of immune activation, and immune status of TME. One key biomarker is the tumor’s capacity to absorb and retain PS, which can be evaluated through imaging techniques or biopsy, assessing how well PS accumulates within the tumor (164). The effectiveness of PDT is also influenced by the availability of oxygen in the tumor to generate sufficient ROS, since poorly oxygenated (hypoxic) areas would show reduced responsiveness to PDT (165). The presence and composition of TILs, especially CD8^+^ T cells, within a tumor would represent ongoing anti-tumor immune response *in situ*, correlating with more favorable outcomes from cancer treatments, including PDT (166). The levels of cytokines and chemokines, such as IFN-γ, TNF-α, and CXCL10, may reveal an inflammatory TME conducive to the effectiveness of PDT-based immunotherapy (48). Tumors with elevated PD-L1 expression could particularly benefit from combination therapies that include PDT and inhibitors of PD-1/PD-L1, as PDT may modulate PD-L1 expression and boost the efficacy of checkpoint blockade therapy (167). Moreover, a high mutational burden, increasing the neoantigen load, could enhance the immunogenicity of the tumor, potentially leading to a better response to immunotherapy following PDT (168).

#### 2.3.4 Challenges in translating PDT into clinical practice

The advancement of PDT into clinical application will confront several challenges that necessitate further development and optimization of PSs. Currently, innovative approaches are enhancing the safety and efficacy of PDT in treating malignancies, with research focused on three main strategies (169). Firstly, there is an effort to create PSs that can effectively produce ROS even in the low-oxygen environments of tumors, thereby overcoming the limitations posed by TME hypoxia. Secondly, the development of PSs that are selectively activated in TME and the use of tumor-targeted nanocarriers aim to improve the precision of PDT. Lastly, enhancing the penetration depth of the excitation light is critical for the effectiveness of PDT (170). Each therapeutic modality, including combination with chemotherapy or immunotherapy, presents its own set of advantages and challenges. For instance, optimizing the chemotherapy-phototherapy ratio is crucial in combination treatments to maximize efficacy (171). Furthermore, PDT is being explored as an intraoperative adjuvant treatment, offering visual aids to surgeons through PS-stimulated fluorescence and allowing for precise removal and ICD induction (172). However, the standardization of PDT protocols, including the selection of the ideal PS, photo dose, and drug-optical intervals, remains a prerequisite for its widespread application. Additionally, there is a need for specialized irradiation equipment designed for different surgical approaches, such as endoscopic resection or open surgery, to facilitate the integration of PDT into clinical practice (170).

The key obstacles encountered in PDT are: (i) inadequate distribution of photosensitizers (PS) after intravenous administration; (ii) diminished light efficacy in reaching the tumor due to tissue absorption; (iii) depletion of oxygen within the tumor environment, restricting the effectiveness of PDT; (iv) temporary or incomplete damage to tumor blood vessels post-PDT, often followed by the formation or repair of new vessels; and (v) incomplete eradication of the tumor, potentially leading to its recurrence (173). Optimizing nanoparticle (NP) design in PDT is crucial. This includes surface modifications for improved targeting and controlled drug release, as well as developing multifunctional NPs that enable therapeutic and imaging capabilities (174). Accurate dosimetry, considering the tissue penetration and the prevention of normal tissue damage, is essential for delivering precise treatment (172). Addressing potential safety concerns associated with NP-based cancer therapy is another critical aspect. This involves thorough biocompatibility testing, optimization of NP characteristics for safe interaction with biological systems, and controlled drug release to minimize off-target effects and long-term accumulation (175). Designing NPs to specifically target receptors can also reduce the risk of unintended off-target interactions (176). Collaborative efforts in research and development are necessary to advance this promising therapeutic modality (177). Incorporating various drugs into nanoparticle-enabled PDT introduces complexities related to potential toxicity and unexpected side effects. For wider clinical application, thorough preclinical and clinical evaluations should be carried out to maximize efficacy and safety (178).

## 3 Conclusion

In conclusion, the fusion of PDT with the rapidly advancing cancer immunotherapy would make a groundbreaking shift in cancer management strategies. PDT’s unique mechanism of action—leveraging light to activate photosensitizing agents effectively induces ICD in tumor tissues. PDT-induced ICD alone, however, is not sufficient in inducing immune responses that could suppress recurrence and metastasis. On the other hand, cancer immunotherapeutic such as ICIs show limited efficacy in real patient population mostly because of pre-established immunosuppressive TMEs. The combination of PDT with immunotherapeutic agents, such as ICIs, marks a significant stride forward in myriad of preclinical studies. ICIs disrupt the cancer-imposed checkpoints that stifle T-cell activity, thereby unleashing the immune system’s latent potential to recognize and obliterate tumor cells. Preclinical investigations have illuminated the potential of this synergy, demonstrating that the local inflammatory milieu engendered by PDT can be amplified systemically by ICIs, leading to a more robust and durable anti-tumor response. NPs have become versatile tools in drug delivery. In PDT, NPs enabled the targeted delivery of PSs and immune-modulating agents to TME. NP-mediated delivery significantly enhances the effectiveness of PDT, while simultaneously reducing undesired side effects. Further development of tailored NPs for PDT will greatly improve clinical outcomes. Additionally, the amalgamation of PDT with conventional therapies may further predispose TME for more potentiated efficacy of immunotherapy. Looking ahead, the ongoing evolution of PDT in the realm of cancer treatment is marked by the exploration of innovative strategies to overcome its current limitations and maximize its therapeutic potential. Continued research and development are essential to refine PDT protocols, improve PS efficiency in hypoxic environments, via advanced nanoparticle formulations. The future of PDT lies in its ability to be seamlessly integrate into personalized cancer treatment plans, leveraging its unique advantages while addressing its challenges through cutting-edge scientific advancements. In summary, as we continue to untangle the complexities of tumor immunology and TME biology, PDT stands out as a potent partner of fast developing cancer immunotherapy. Accumulated preclinical data in PDT combination with immunotherapy shed bright lights on cancer therapy. But only a limited number of clinical trials have been carried out so far, which holds back the therapeutic application of PDT/immunotherapy combination to real patients. Given the active and widespread application of RT in combination with immunotherapy in clinical settings, PDT will come into the cancer immunotherapy arena in the near future.

## Author contributions

JT: Writing – original draft. VV: Writing – original draft. I-KP: Writing – review & editing. SL: Writing – review & editing. JR: Conceptualization, Funding acquisition, Investigation, Supervision, Validation, Writing – review & editing.
